# Time Circular Birefringence in Time-Dependent Magnetoelectric Media

**DOI:** 10.1038/srep13673

**Published:** 2015-09-02

**Authors:** Ruo-Yang Zhang, Yan-Wang Zhai, Shi-Rong Lin, Qing Zhao, Weijia Wen, Mo-Lin Ge

**Affiliations:** 1 Theoretical Physics Division, Chern Institute of Mathematics, Nankai University, Tianjin, 300071, China; 2 Department of Physics, The Hong Kong University of Science and Technology, Clear Water Bay, Hong Kong, China; 3 School of Physics, Beijing Institute of Technology, Beijing, 100081, China

## Abstract

Light traveling in time-dependent media has many extraordinary properties which can be utilized to convert frequency, achieve temporal cloaking, and simulate cosmological phenomena. In this paper, we focus on time-dependent axion-type magnetoelectric (ME) media, and prove that light in these media always has two degenerate modes with opposite circular polarizations corresponding to one wave vector 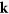, and name this effect “time circular birefringence” (TCB). By interchanging the status of space and time, the pair of TCB modes can appear simultaneously via “time refraction” and “time reflection” of a linear polarized incident wave at a time interface of ME media. The superposition of the two TCB modes causes the “time Faraday effect”, namely the globally unified polarization axes rotate with time. A circularly polarized Gaussian pulse traversing a time interface is also studied. If the wave-vector spectrum of a pulse mainly concentrates in the non-traveling-wave band, the pulse will be trapped with nearly fixed center while its intensity will grow rapidly. In addition, we propose an experimental scheme of using molecular fluid with external time-varying electric and magnetic fields both parallel to the direction of light to realize these phenomena in practice.

For general linear nondispersive bianisotropic media, the constitutive relations are


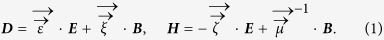


The tensors 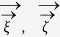 correspond to the magnetoelectric (ME) cross polarizations. A ME medium satisfying 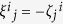 is reciprocal, *e.g*. chiral medium, otherwise it is nonreciprocal. The nonreciprocal ME effect was first discovered in Cr_2_O_3_^1^,^2^,^3^, and has attracted wide attention both in condensed matter physics^4^,^5^,^6^,^7^,^8^,^9^,^10^,^11^ and in optics^12^,^13^,^14^,^15^,^16^,^17^,^18^,^19^,^20^,^21^,^22^,^23^. It has been shown that a nonreciprocal ME medium with nonzero 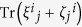 can separate a real term Θ from the ME coupling^12^,^13^,^14^. If we are only concerned with this term, the two ME coefficients reduce to isotropy: 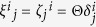. Then the Maxwell equations can be expressed as the axion-like form^7^,^24^ with the virtual electric displacement 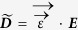 and the virtual magnetic field 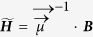 excluding the electric and magnetic cross polarizations. By redefining a virtual excitation tensor 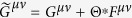 constructed from the virtual fields: 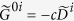, 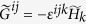, the lagrangian density in the isotropic ME media can be written as same as the one in axion electrodynamics^7^,^8^,^9^:


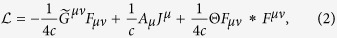


where 
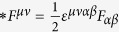
 is the Hodge dual of *F*_*αβ*_. In Eq. (2), the last term 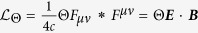 just corresponds to the axion coupling, and 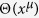 corresponds to the axion field. Correspondingly, the 4-D Maxwell equation also holds the axion-like form 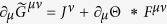. Since ***E*** is a polar vector while ***B*** is an axial vector, Θ must be a pseudoscalar to guarantee that the lagrangian density is a Lorentz scalar.

Axion was originally proposed as a hypothetical elementary particle^25^, while it won great interests in condensed matter physics recently because of the significant discovery that an effective quantized axion field can be induced in topological insulators when time reversal symmetry is weakly broken^7^,^8^,^9^,^10^,^11^. Actually, since Θ is a pseudoscalar, the axion-type ME coupling only exists in the systems where both the time reversal (*T*) and the parity (*P*) symmetries are broken but the combined *PT* symmetry is held^3^. There is no visible effect for light traveling in globally constant axion field, however, a Kerr or Faraday rotation can be detected for lights reflected or refracted by the surface of an axion medium^7^,^20^,^21^,^22^, which essentially originates from the sudden change of Θ at the spatial interface^7^. Noteworthy, a type of circular birefringence, known as Carroll-Field-Jackiw (CFJ) birefringence, can emerge in Chern-Simons modified electrodynamics^26^. And Y. Itin proved that the CFJ birefringence can be alternatively caused by a space-and-time-dependent axion field in geometric optics approximation^27^,^28^. The CFJ birefringence is generally anisotropic in space, whereas it reduces to isotropy when the 4-gradient 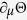 is timelike, *i.e*. the axion field only changes with time.

Light traveling in time-dependent media has many extraordinary properties which can be utilized to achieve frequency conversion^29^,^30^, temporal cloaking^31^,^32^,^33^,^34^, and to simulate cosmological phenomena^35^,^36^ *etc*. In this paper, we focus on time-dependent axion-type ME media, and prove that light in these media always has two oppositely circularly polarized modes corresponding to one wave vector ***k*** but not limited to geometric optics approximation. The key idea of this paper is to interchange the status of space and time. We will show that the pair of TCB modes can appear simultaneously via the “time refraction” and “time reflection” of a linearly polarized incident wave at a time-discontinuous interface of the ME media. The superposition of two TCB modes causes the “time Faraday effect” which is a novel effect as a temporal counterpart of the ordinary spatial Faraday effect or optical activity. Further discussions about the propagating velocities of energy and information for TCB modes and about the time refraction and reflection of Gaussian pulse at time interfaces in ME media are also provided. Furthermore, we put forward an experimental scheme to generate the effective time-dependent axion-type ME media controlled by time-varying external electric field 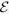 and magnetic field 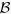 parallel to each other which offers a practical way to realize the novel phenomena predicted in this paper.

## Results

### Time circular birefringence and time Faraday effect

In time-dependent axion-type ME media, the magnetic induction obeys the wave equation


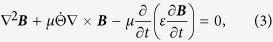


where the dot over Θ denotes the derivative with respect to time, and *ε*, *μ*, Θ are all functions of time in general. While the *P* and *T* symmetries are both broken in Eq. (3), the combined *PT* symmetry is preserved. Considering the class of solutions 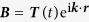 with a constant wave vector ***k***, the temporal part satisfies 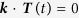 due to 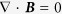. Therefore, the temporal part can be further separated into two independent circularly polarized portions 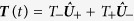 obeying the following equations respectively


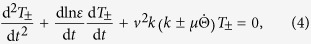


where 
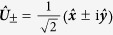
 are the circularly polarized bases with choosing the direction of ***k*** to be *z* axis, and 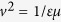. As a result, there always exists a pair of circularly birefringent modes *T*_±_ for a given wave vector ***k*** in time-dependent axion-type media: 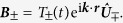 We call this effect the time circular birefringence (TCB). If 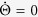, the two distinct equations of *T*_±_ reduce to an identical one, and the birefringent phenomenon vanishes. Thereby TCB is entirely induced by the time varying axion field. In addition, TCB happens in isotropic media, thus it is different from both the ordinary birefringence in uniaxial or biaxial crystals and the ME Jones birefringence^37^,^38^,^39^,^40^ which are all caused by the anisotropy of materials. TCB is also different from the optical active circular birefringence (OACB), because TCB is generated from the temporal nonhomogeneity of the nonreciprocal ME media but OACB is a reciprocal magnetoelectric effect originating from the chirality of molecules.

For traditional birefringent effects, two different wave numbers *k* correspond to one frequency. One can realize the two birefringent states just via shooting a beam onto a birefringent medium subject to the temporal-phase-matching condition 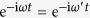 at the spatial interface. However, the temporal parts *T*_±_(*t*) of the pair of TCB modes corresponding to a fixed wave number are different, and accordingly could not match the temporal phase factor of the incident wave simultaneously. This difference gives rise to a handicap for realizing this pair of circular polarized states in practice. To overcome this difficulty, we think up the idea of “time discontinuous media” by analogy with the “spatial discontinuous media” used in traditional birefringent systems, then the spatial phase factor, 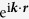, should be matched at time interfaces. Considering a time-dependent medium *ε*(*t*), *μ*(*t*), Θ(*t*) discontinuous at a time interface *t*_0_, we can get the temporal boundary conditions of electromagnetic fields by integrating Maxwell equations over an infinitesimal time interval across *t*_0_^41^,^42^:


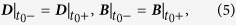


while ***E*** and ***H*** are generically discontinuous at the time interface.

**Figure 1:**
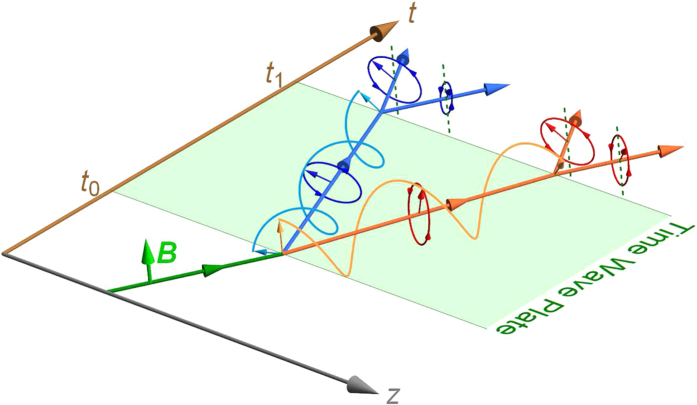
Illustration of time refraction, time reflection and time Faraday rotation for a linearly polarized light incident upon a time wave plate with time-dependent ME coefficient Θ = *βt*/*μ*_1_(*t*_0_ < t t1). At *t*_0_, the wave splits into a time refracted part and a time reflected part. The two parts are both elliptically polarized, but their major axes rotate with time. After the second interface *t*_1_, the polarization axes of the four outgoing waves have angular differences with respect to the polarization of the incident wave.

Just as spatial optical wave plate devices, we analyze light propagating in a “time wave plate” with piecewise medium parameters: *ε*_0_, *μ*_0_, Θ_0_ are constant when *t* < *t*_0_; *ε*_1_(*t*), *μ*_1_(*t*), Θ_1_(*t*) are some continuous functions when *t*_0_ < *t* < *t*_1_; *ε*_2_, *μ*_2_, Θ_2_ are also constant when *t* > *t*_1_, as shown in Fig. 1. For a linearly polarized incident wave 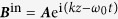 with 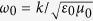 and 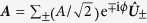, the wave will become the sum of the two TCB modes 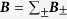 at *t*_0_. Moreover, there always exist two linearly independent solutions for Eq. (4) which are complex conjugates of each other: 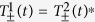, then the general solution of Eq. (4) is their superposition: 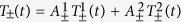, and the two TCB states can be further separated as 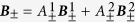. It can be proved that the momentums of the two branches 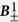 and 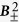 are always in opposite directions, i.e. one branch always propagates along the incident direction (for convenience, let it be 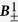), while the other (let it be 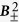) is always along the opposite. As a result, 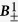 and 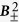 are exactly the “time refraction” and “time reflection” of the corresponding TCB modes at the time interface *t*_0_ (see the supplementary information for more discussions).

A simplified case is 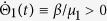, and *ε*_1_, *μ*_1_ are both constant. Then the TCB modes are identical with the CFJ modes obtained in geometric optics approximation^26^,^27^,^28^, therefore the light splits into two plane waves


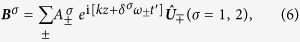


as 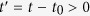. The dispersion relations of two TCB modes are 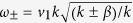, and the coefficients determined by the temporal boundary conditions are


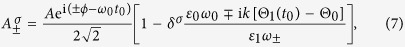


with 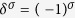. According to the dispersion relations, the two TCB modes ***B***_±_ both have a forbidden band of *k* for traveling waves: 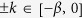. Outside the forbidden band, ***B***^1^ travels along the incident direction, i.e. it is the time refraction, and ***B***^2^ is the time reversal of ***B***^1^. However, a wave should not propagate backwards through time. The practical observable is its real part which propagates opposite to the incident direction in space, therefore, ***B***^2^ is actually the time reflection. Without loss of the physical generality, a further simplification will applied in the following: *ε*_0_ = *ε*_1_ = *ε*_2_, *μ*_0_ = *μ*_1_ = *μ*_2_, Θ_1_(*t*_0_) = Θ_0_, and Θ_1_(*t*_1_) = Θ_2_, *i.e*. the medium is continuous at *t*_0_ and *t*_1_ but 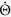 is still discontinuous.

**Figure 2:**
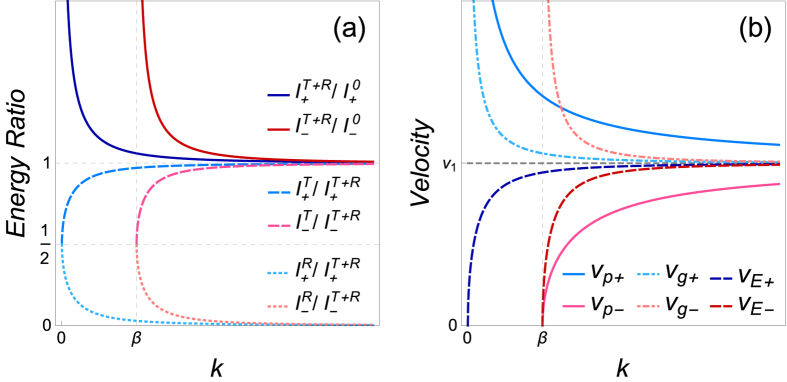
(**a**) Ratios of total light intensity of refraction and transmission to the incident light intensity 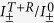, modified transmissivity 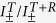, and modified reflectivity 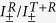 corresponding to the two TCB modes respectively are shown as functions of wave number *k* (see the supplementary information for more details). (**b**) Phase velocities *v*_p±_, group velocities *v*_g±_, energy transport velocities *v*_E±_ and front velocity 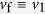 of the two TCB modes versus *k*.

The time dependence of media destructs the symmetry of time translation, therefore, the energy of the electromagnetic field is not conserved in general. On the other hand, the lagrangian of time dependent media shown in Eq. (2) is invariant under spatial translation, so the apparent electromagnetic momentum 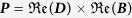 must be conserved. Typically, the energy of incident wave does not equal to the total energy of the time refracted and reflected waves at the time interfaces of a time wave plate (see Fig. 2(a)), whereas the incident apparent momentum equals to the resultant momentum of the reflected and refracted waves: 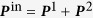 (see the supplementary information for general proof). From a photonic point of view, the nonconservation of energy indicates 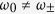, while the conservation of momentum insures ***k***^in^ = ***k***^1^ = ***k***^2^ at time interfaces. This fact is different from the case of ordinary refraction and reflection at a spatial interface of two media, in which the energy is conserved, but the normal momentum to the spatial interface isn’t conserved because the discontinuity of the media breaks the symmetry of spatial translation.

As shown in Eq. (6), the refracted and reflected waves both have two circularly polarized components with different frequencies *ω*_±_. The superposition of the two components gives rise to the time Faraday rotation (TFR), namely, the refracted and reflected waves can be rewritten as a sole polarized wave respectively


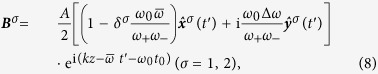


with the time dependent bases


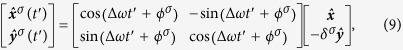


where 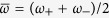, 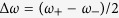, 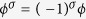. So both the time refracted and reflected waves can be regarded as generic elliptically polarized plane waves propagating with the frequency 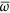, but their polarization ellipses rotate with angular velocity Δ*ω*, *i.e*. the TFR. Because of the *PT* symmetry, the refracted and reflected waves rotate in same chirality with respect to their respective propagating directions. Unlike ordinary magneto-optical Faraday effect or optical activity which both refer to the polarization of a wave changing circularly in its propagating direction, the TFR wave has a unique polarization in the whole space at any fixed time point, however, the polarization rotates with time. Note that the Faraday effect caused by two opposite circularly polarized CFJ waves was also discussed in Ref. 26. However, their effect is still a spatial Faraday rotation, *i.e*. the two superposed CFJ waves have same frequency *ω* but different *k* and the rotating angle changes with traveling distance, therefore the TFR caused by the time refraction and time reflection is entirely a novel effect distinct form their discussion.

At the second time interface *t*_1_ of the time wave plate, the secondary time refraction and reflection occur. Then the beam will split into four elliptically polarized branches, all of which can be written by


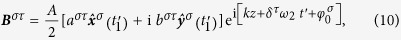


where 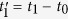, and 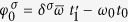. The superscript *στ* distinguishes the four branches: *στ* = 11, 12 denote, respectively, the secondary refraction and reflection of the first refracted wave, and *στ* = 22, 21 denote the secondary refraction and reflection of the first reflected wave respectively. Eq. (10) shows that the Faraday rotating angle of the polarization ellipses of the four secondary branches is 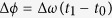 as the waves pass through the time wave plate (see Fig. 1). And in terms of the boundary conditions at 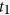, the relative lengths of the two polarized axes satisfy


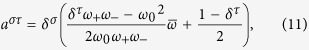



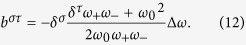


### Velocities of TCB modes

The phase velocities and the group velocities of two TCB modes are, respectively,


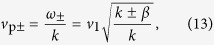



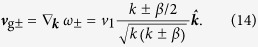


As noted in Ref. 26, 27, 28, the two phase velocities meet 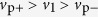, and the two group velocities meet 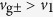. For the axion field in vacuum, 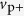 and 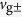 always exceed the speed of light *c* in vacuum. Though *v*_1_ < *c* in real media, 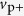 and 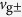 will be still superluminal when 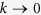 for 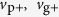 and 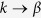 for 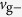. However, neither phase velocity nor group velocity represents the true velocity of energy or information transfer, therefore the superluminal effects of these two types of velocities do not violate the causality and have been observed in various experiments^43^,^44^,^45^. By means of the average Poynting vector and energy density over a period, we also can calculate the energy transport velocities of the two TCB states


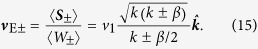


On the contrary to the group velocities, 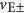 are always less than *v*_1_. Moreover, we prove that the front velocity *v*_f_ (the velocity of wave front which represents the speed of information propagation) of the two TCB modes is precisely *v*_1_, when only concerning the dispersion caused by the constant rate *β* of the ME coefficients but regardless of the dispersion of *ε*, *μ*, *β* with respect to wave number *k* (the detailed derivation is given in the supplementary information). Therefore, neither energy nor information of TCB modes propagates superluminally. The comparison of four types of velocities is shown in Fig. 2 (b).

### Gaussian pulse traversing a time interface

The plane wave solutions we have discussed are widespread in the whole space. However, the time wave plate made of time dependent media should only have a finite scale in practice. We accordingly need to analyze the propagation of wave packages with finite length. Consider a Gaussian pulse 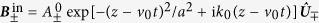 with left or right circular polarization and width *a* incident onto the time interface *t*_0_ of a time wave plate. Here, we still only concern the dispersion caused by *β*. Taking account of the temporal boundary conditions, we obtain the magnetic fields, for *t* > *t*_0_,


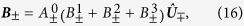


where 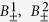 denote the time refraction and reflection parts respectively, and 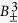 denotes the non-traveling wave part. The three parts of ***B***_***−***_ take the forms


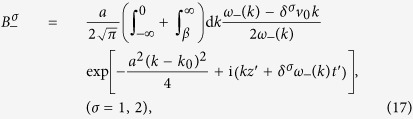



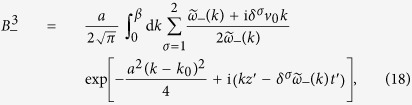


with 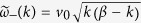 and 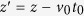. And the three parts of ***B***_+_ have similar expressions.

**Figure 3:**
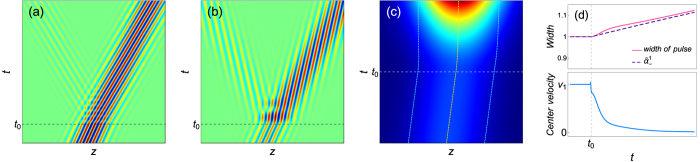
Magnetic field patterns of circularly polarized states (a) *B*_+_ and (b) B_−_ in spacetime for a corresponding polarized Gaussian pulse incident onto the time interface *t*_0_, whose *k* spectrum mainly concentrates in the traveling-wave band, namely *k*_0_ − ≫ 2/*a*. (**c**) 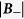 of a pulse whose *k* spectrum mainly concentrates in the non-traveling- wave band, namely 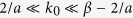. In this case, the pulse is nearly trapped while its intensity increases rapidly. The yellow dashed curve and the two light-blue dashed curves trace the center of the pulse and the edges of the pulse respectively. (**d**) The width (scale of 1 to the original width 2*a*) and the center velocity of the pulse varying with time.

For the situation 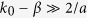, the non-traveling wave part 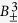 can be neglected, and the range of integration in Eq. (17) can approximate to −∞ to ∞. In addition, we expand *ω*_±_(*k*) near the center wave number *k*_0_ in a Taylor series


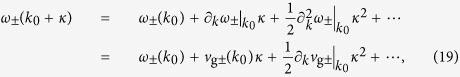


and neglect the high order terms (order ≥3), then the refracted and reflected pulses have the approximations:


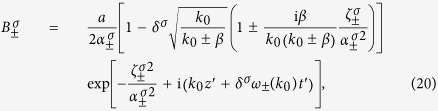


where 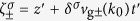 is the relative coordinate with respect to the center of the wave package, and 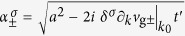. The time refractions and time reflections for two different circularly polarized pulses are shown in Fig. 3(a,b). Actually, this approximation is valid only when 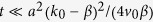, because 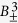 increases exponentially. However, as 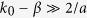, the upper bound of time could be a long period. According to Eq. (20), the term proportional to i*β* is extremely small in the main range of the pulses 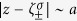. Omitting this term, it is clear that the pulse propagates with group velocity *v*_g±_, and the dispersion of *v*_g±_ induces the pulse width to change with time.

For another particular case 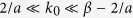, the traveling parts of refraction and reflection 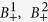 shown in Eq. (20) still offer the major contribution to ***B***_+_. However, ***B***_−_ mainly concentrates in the non-traveling part, ignoring the refraction and reflection parts 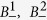 is thus reasonable, and the approximate solution reads


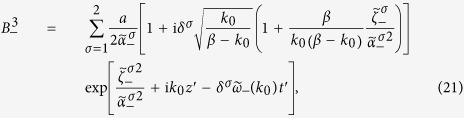


with 
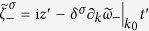
, 
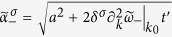
. Fig. 3(c) shows the pattern of 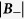 as the pulse traversing the time interface. Fig. 3(d) plots the velocity of the pulse center and the width of the pulse (defined as the distance between the two edges where 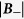 equal to 1/e times 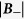 at the center of the pulse) changing with time. Consequently, the pulse keeps nearly fixed center after traversing the time interface, while its intensity increases with the magnitude about exp (*t*^2^). The width of the pulse increases with time, and it can be characterized by 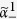 approximately as shown in Fig. 3(d).

### Experimental design

Considering a fluid in the presence of external electric and magnetic fields, the multipolar polarizations induced by external electric or magnetic fields can cause the fluid to be anisotropic and lead to Kerr effect or Cotton-Mouton effect. More specially, a parallel pair of external electric field 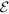 and magnetic field 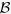 will induce the Jones birefringence for a light beam propagating perpendicularly to the direction of the fields^37^,^38^,^39^,^40^. The Jones birefringence has been shown to be a bianisotropic effect^39^. For symmetric analysis, the external electric field 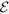 is *P* odd, and the external magnetic field 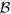 is *T* odd, but the parallelism of the two fields protects the combined *PT* symmetry. This fact indicates the existence of the axion type ME coupling as we have mentioned. The ME coupling tensor of molecules can be expanded with respect to the external fields


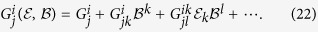


The coefficients of each order are determined by solving the time-dependent perturbation of the molecular hamiltonian^40^. The Boltzmann average over all orientations of diamagnetic molecules yields^37^,^40^


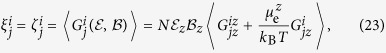


where the external fields are supposed to parallel *z* axis, *N* is the number density of molecules, 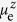 is the *z* component of the permanent molecular electric dipole moment, *k*_B_ is the Boltzmann constant, and *T* is temperature. Since the system is symmetric with respect to *z* axis, the medium should retain isotropic in the *x* − *y* plane and has a uniaxial ME tensor 
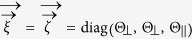
. Thus a beam propagating perpendicularly to *z* axis has two Jones birefringent eigenmodes, linearly polarized along the ±*π*/4 directions with respect to *z* axis respectively, with the difference of refractive indexes 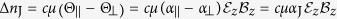
39. However, if a transverse polarized light travels along *z* axis, *i.e*. parallel to the external fields, it will experience the isotropic axion-type ME coupling 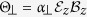. In terms of isotropic average^37^, the ME coefficient in *x* − *y* plane, is


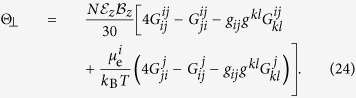


**Figure 4:**
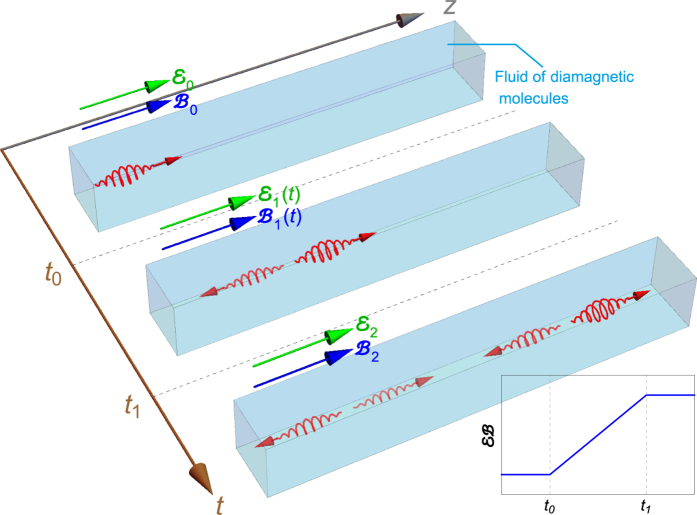
Illustration of circularly polarized pulse traveling in a fluid of diamagnetic molecules located in time-dependent external electric field 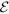 and magnetic field 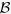 both parallel to the propagating direction of the pulse. For the three-piece product 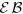, the fluid acts as a time wave plate. At *t*_0_, the incident pulse splits into a refracted one and a reflected one. At *t*_1_, the two pulses further split into four.

As a result, the effective axion field can be controlled via the external electric and magnetic fields. If the product of the external fields 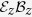 changes with time, we could observe the TCB and correlated phenomena predicted in this paper. The schematic illustration are shown in Fig. 4.

In principle, the TCB, as well as the ME coupling, caused by the time-varying external fields can arise in all media, while its magnitude is characterized by 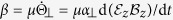. Supposing the product of the fields varies linearly with time, the magnitude is determined by two parts, one is the intrinsic property of the medium *α*_^_, the other is the rate of field change 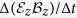. In the first order approximation, the frequencies and the phase velocities of the two TCB modes are 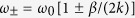 and 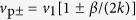 respectively. And the refractive-index difference of the two TCB modes is


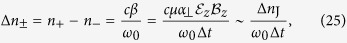


with the assumption that the product of the external fields increases linearly from 0 to the the final value 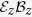 in the time interval Δ*t*. Here, the symbol “~” means the quantities of two sides have the same order of magnitude, since *α*_^_ and 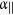 are generically in the same order.

According to the experimental results in Ref. 38,39, molecules with a low-lying strong charge transfer transition of approximately octupolar symmetry and a permanent electric dipole moment will have relative large ME coupling. In this experiment, the Jones birefringence are observed in three typical molecular liquids, namely methylcyclopentadienyl-Mn-tricarbonyl, cyclohexadienyl-Fe-tricarbonyl, and Ti-bis(ethyl-acetoacetato) diisopropoxide, with the magnitude about Δ*n*_J_ ~ 10^−11^ under the parameters 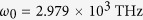 (HeNe laser), 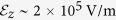, 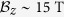 at room temperature and 1 atm. Adopting these experimental parameters and assuming the time interval of field change Δ*t* ~ 10^−9^ s (the characteristic frequency of the external fields is equivalent to GHz), we can estimate the refractive-index difference of the two TCB modes 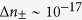. On the other hand, previous experiments for small birefringence measurements have achieved the sensitivity 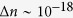 via the metrology of high finesse resonant cavity^46^,^47^,^48^, to measure the TCB effect is accordingly feasible. Since the group velocities of the two TCB modes are nearly equal 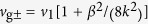 for small *β*, we can ignore the central separation of two superposed TCB pulses during the time interval Δ*t* and regard them as a single pulse with the TFR 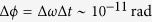 which is large enough for detection as a 10^−13^ rad resolution of phase shift has been achieved experimentally^49^.

If the external fields are both parallel to the propagating direction of the pulse rigorously, no other birefringent effects that can disturb the observation of TCB, *e.g*. Kerr or Cotton-Mouton effects, would arise. However, the time dependence of the external fields will induce fields in the *x* − *y* plane inevitably. Supposing only 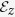 changes with time but 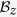 is constant, the linearly varying 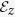 induces an eddy magnetic field 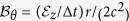 around *z* axis, and 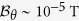 in the area of *r* < 10^−2^ m which is thus small enough to be ignored. For experimental setup, a big challenge is to precisely control the external fields. Theoretically, the external fields at any locations should change simultaneously in the laboratory reference system, namely the variation of 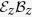 at different points is spacelike, since the effective axion field Θ_^_ only depends on time. In practice, the speed of light in the media is less than vacuum, thus the prerequisite could be relaxed into that the fields begin to change before the pulse arrives. If there is a slow-light system with strong ME coupling *α*_^_, then the technical requirement could be largely reduced.

## Conclusion

To summarize, we demonstrate that light with a certain wave vector ***k*** always corresponds to a pair of circularly polarized modes, *i.e*. the TCB modes, in time-dependent axion-type ME media. We study the time refraction and time reflection of plane waves and Gaussian pulses traveling in this type of media, and predict the time Faraday effect as a consequence of the superposition of the two TCB modes. We also propose a scheme to realize TCB in practice. According to our estimations with the realistic parameters, the magnitude of TCB is observable via existing experimental techniques. As the significance but difficulty for detecting axion particles, our proposal offers an alternative way to simulate and study the interaction of light with time-dependent axion field. On the other hand, by exchanging the status of space and time, we foresee that various effects in space-dependent media would have their temporal counterparts in time-dependent media for not only electromagnetic fields but also all kinds of waves. We hope our work could inspire more research in this novel area.

## Additional Information

**How to cite this article**: Zhang, R.-Y. *et al*. Time Circular Birefringence in Time-Dependent Magnetoelectric Media. *Sci. Rep*. **5**, 13673; doi: 10.1038/srep13673 (2015).

## Supplementary Material

Supplementary Information
