# Supplementary material for: Time Circular Birefringence in Time-Dependent Magnetoelectric Media
Source: Sci Rep. 2015 Sep 2;5:13673. doi: 10.1038/srep13673 (PMC4556965; doi:10.1038/srep13673)
Supplement: Supplementary Information [file srep13673-s1.pdf]

Supplementary Information: Time Circular Birefringence in Time-Dependent Magnetoelectric Media

Ruo-Yang Zhang,[1,2] Yan-Wang Zhai,[3] Shi-Rong Lin,[3] Qing Zhao,[3] Weijia Wen,[2] and Mo-Lin Ge[1,3]

[1] *Theoretical Physics Division, Chern Institute of Mathematics, Nankai University, Tianjin, 300071, China*
[2] *Department of Physics, The Hong Kong University of Science and Technology, Clear Water Bay, Hong Kong, China*
[3] *School of Physics, Beijing Institute of Technology, Beijing, 100081, China*

In this supplementary information, we will give a further discussion about the time refraction and time reflection of TCB modes in arbitrary time dependent axion-type ME media, and will give the derivation of the wave front velocity $v_{\mathrm{f}} = v_1$ of TCB modes with the simplified dispersion relation $\omega_\pm = v_1 k\sqrt{(k \pm \beta)/k}$ in detail.

Time refraction and time reflection of TCB modes in general conditions

The two TCB modes in time-dependent axion-type ME media take the form $\boldsymbol{B}_\pm = T_\pm(t)\mathrm{e}^{\mathrm{i}kz}\hat{\boldsymbol{U}}_\mp$, where the temporal parts $T_\pm(t)$ satisfy the following equation:

$$\frac{\mathrm{d}^2 T_\pm}{\mathrm{d}t^2} + \frac{\mathrm{d}\ln\varepsilon}{\mathrm{d}t}\frac{\mathrm{d}T_\pm}{\mathrm{d}t} + v^2 k(k \pm \mu\dot{\Theta})T_\pm = 0. \tag{S1}$$

where $\varepsilon,\ \mu,\ \Theta$ are all functions of time in general. For simplicity, we demand all the parameters in Eq. (S1) are real. According to the Maxwell equations and the constitutive relations, the other three electromagnetic vectors of the corresponding TCB modes read

$$\boldsymbol{D}_\pm = \Theta\boldsymbol{B}_\pm \pm \frac{\varepsilon}{k}\dot{\boldsymbol{B}}_\pm, \qquad \boldsymbol{E}_\pm = \pm\frac{1}{k}\dot{\boldsymbol{B}}_\pm, \quad \boldsymbol{H}_\pm = \frac{1}{\mu}\boldsymbol{B}_\pm \mp \frac{\Theta}{k}\dot{\boldsymbol{B}}_\pm. \tag{S2}$$

Arbitrary two linearly independent solutions of linear Eq. (S1) can be regarded as the bases of its solution space. Supposing $g(t)$ and $h(t)$ are two independent real solutions of (S1), then other two solutions that are complex conjugates of each other can be constructed:

$$T^1_\pm(t) = g(t) + \mathrm{i}h(t) = \rho_\pm(t)\mathrm{e}^{\mathrm{i}\psi_\pm(t)}, \quad T^2_\pm(t) = g(t) - \mathrm{i}h(t) = \rho_\pm(t)\mathrm{e}^{-\mathrm{i}\psi_\pm(t)}, \tag{S3}$$

where $\rho_\pm(t)$ and $\psi_\pm(t)$ are the amplitude and the polar angle of $T^1(t)_\pm$. $T^1_\pm(t)$ and $T^2_\pm(t)$ are also a set of bases of the solution space. Therefore, $\boldsymbol{B}_\pm$ can always separate into two parts $\boldsymbol{B}_\pm = \boldsymbol{B}^1_\pm + \boldsymbol{B}^2_\pm$ with

$$\boldsymbol{B}^1_\pm = A^1_\pm\rho_\pm(t)\mathrm{e}^{\mathrm{i}(kz+\psi_\pm(t))}, \qquad \boldsymbol{B}^2_\pm = A^2_\pm\rho_\pm(t)\mathrm{e}^{\mathrm{i}(kz-\psi_\pm(t))}. \tag{S4}$$

Consider a linearly polarized incident plane wave $\boldsymbol{B}^{\mathrm{in}} = \boldsymbol{A}\mathrm{e}^{\mathrm{i}(kz-\omega_0 t)} = \sum_\pm (A/\sqrt{2})\mathrm{e}^{\mathrm{i}(kz-\omega_0 t\pm\phi)}\hat{\boldsymbol{U}}_\mp$, as $t < t_0$, with $\omega_0 = k/\sqrt{\varepsilon_0\mu_0}$ and $\boldsymbol{A} = \sum_\pm (A/\sqrt{2})\mathrm{e}^{\mp\mathrm{i}\phi}\hat{\boldsymbol{U}}_\pm$, where ϕ is the polarized angle with respect to x axis. After the wave passes through the time interface t_0 of the time wave plate, the wave becomes the superposition of the two TCB modes $\boldsymbol{B} = \sum_\pm \boldsymbol{B}_\pm$, and the two TCB modes can be further separate into two independent parts given in Eq. (S4). In terms of the temporal boundary conditions, the coefficients of the two parts can be determined

$$A^\sigma_\pm = \frac{A^{\mathrm{in}}_\pm \mathrm{e}^{\mathrm{i}(\pm\phi-\omega_0 t_0+\delta^\sigma\psi_\pm(t_0))}}{2\rho_\pm(t_0)\varepsilon_1(t_0)\dot{\psi}_\pm(t_0)}\delta^\sigma\Big[\big(\varepsilon_0\omega_0 - \varepsilon_1(t_0)\omega^\sigma_\pm(t_0)\big) + \mathrm{i}k\big(\Theta_1(t_0) - \Theta_0\big)\Big], \quad (\sigma = 1, 2), \tag{S5}$$

where $\omega^\sigma_\pm(t) = \mathrm{i}\frac{\mathrm{d}}{\mathrm{d}t}\ln T^\sigma_\pm(t) = \frac{\mathrm{d}}{\mathrm{d}t}[\delta^\sigma\psi_\pm(t) + \mathrm{i}\ln\rho_\pm(t)]$, and $A^{\mathrm{in}}_\pm$ is the amplitude of corresponding circularly polarized incident wave, for the case of linearly polarized incident wave, $A^{\mathrm{in}}_\pm = A/\sqrt{2}\mathrm{e}^{\mp\mathrm{i}\phi}$. If $\dot{\Theta}_1(t) \equiv \beta/\mu_1 > 0$, and $\varepsilon_1,\ \mu_1$ are constant, Eq. (S5) reduces to the simplified expression given in Eq. (7) of the main text.

Because the lagrangian does not contain spatial coordinates explicitly, i.e. the system is invariant under spatial translation, according to Noether's theorem, the conservation of momentum of the system can be expressed as

$$\frac{\partial}{\partial t}(\boldsymbol{D}\times\boldsymbol{B}) + \nabla\cdot\left[\frac{1}{2}\big(\boldsymbol{D}\cdot\boldsymbol{E} + \boldsymbol{B}\cdot\boldsymbol{H}\big)\overset{\rightrightarrows}{I} - \boldsymbol{D}\boldsymbol{E} - \boldsymbol{H}\boldsymbol{B}\right] = 0, \tag{S6}$$

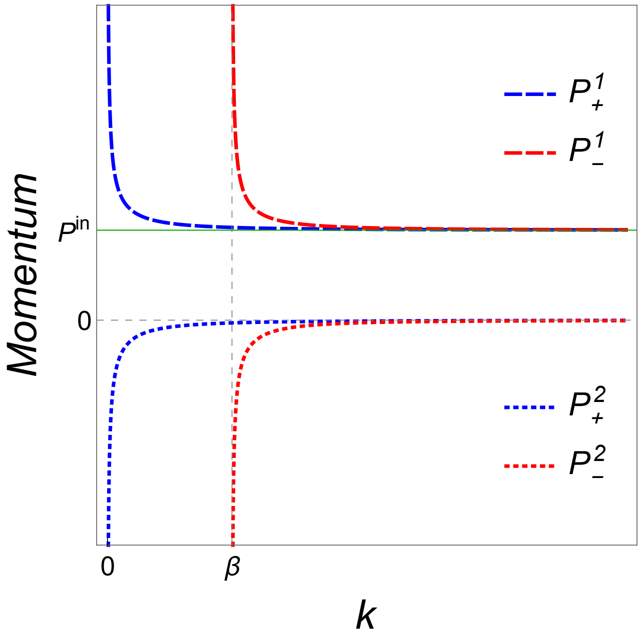

FIG. S1. Apparent momentum densities of the time refracted and time reflected parts of the two TCB modes versus k for the simplified case $\dot{\Theta}_1(t) \equiv \beta/\mu_1 > 0$, and $\varepsilon_1 \equiv \varepsilon_0,\ \mu_1 \equiv \mu_0$.

where $\boldsymbol{P} = \boldsymbol{D}\times\boldsymbol{B}$ is the apparent momentum density of electromagnetic fields, and $\vec{\vec{M}} = \frac{1}{2}\big(\boldsymbol{D}\cdot\boldsymbol{E}+\boldsymbol{B}\cdot\boldsymbol{H}\big)\vec{\vec{I}}-\boldsymbol{D}\boldsymbol{E}-\boldsymbol{H}\boldsymbol{B}$ is the Maxwell stress tensor. Since the TCB modes are transverse with respect to $\boldsymbol{k}$ and their spatial parts merely vary with z, we have $\partial\boldsymbol{P}_\pm/\partial t = -\nabla\cdot\vec{\vec{M}}_\pm = 0$. Therefore, the momentum density $\boldsymbol{P}_\pm$ should be constant.

For $t > t_0$, the momentum density is $\boldsymbol{P}_\pm = \mathfrak{Re}(\boldsymbol{D}^1_\pm + \boldsymbol{D}^2_\pm) \times \mathfrak{Re}(\boldsymbol{B}^1_\pm + \boldsymbol{B}^2_\pm) = \boldsymbol{P}^1_\pm + \boldsymbol{P}^2_\pm + \boldsymbol{P}^{\text{cross}}_\pm$, where $\boldsymbol{P}^1_\pm = \mathfrak{Re}(\boldsymbol{D}^1_\pm) \times \mathfrak{Re}(\boldsymbol{B}^1_\pm)$ and $\boldsymbol{P}^2_\pm = \mathfrak{Re}(\boldsymbol{D}^2_\pm) \times \mathfrak{Re}(\boldsymbol{B}^2_\pm)$ are the momentums of the two independent parts respectively, and $\boldsymbol{P}^{\text{cross}}_\pm = \mathfrak{Re}(\boldsymbol{D}^1_\pm) \times \mathfrak{Re}(\boldsymbol{B}^2_\pm) + \mathfrak{Re}(\boldsymbol{D}^2_\pm) \times \mathfrak{Re}(\boldsymbol{B}^1_\pm)$ is the cross term. Substituting Eq. (S2) and Eq. (S4) into the momentum densities, we obtain the cross term is alway zero $\boldsymbol{P}^{\text{cross}}_\pm \equiv 0$ and $\boldsymbol{P}^\sigma_\pm = \delta^\sigma \frac{1}{2k}\varepsilon_1(t)\dot{\psi}_\pm(t)\rho_\pm(t)^2 \left|C^\sigma_\pm\right|^2 \hat{\boldsymbol{z}}$ ($\sigma = 1, 2$). It is easy to check that $F = \varepsilon_1(t)\dot{\psi}_\pm(t)\rho_\pm(t)^2$ is a first integral of the ordinary differential equation (S1). Actually, $F = \varepsilon_1(t)[\dot{g}(t)h(t) - g(t)\dot{h}(t)]$ according to Eq. (S3). Since $g(t)$ and $h(t)$ are both the solutions of (S1)

$$\varepsilon_1\ddot{g} + \dot{\varepsilon}_1\dot{g} + \varepsilon_1 v^2 k(k \pm \mu\dot{\Theta})g = 0, \tag{S7a}$$

$$\varepsilon_1\ddot{h} + \dot{\varepsilon}_1\dot{h} + \varepsilon_1 v^2 k(k \pm \mu\dot{\Theta})h = 0, \tag{S7b}$$

computing (S7a)$\cdot h-$(S7b)$\cdot g$ yields $\frac{\mathrm{d}}{\mathrm{d}t}F = 0$, so $F \equiv \varepsilon_1(t_0)\dot{\psi}_\pm(t_0)\rho_\pm(t_0)^2$. Then we obtain

$$\boldsymbol{P}_\pm = \boldsymbol{P}^1_\pm + \boldsymbol{P}^2_\pm = \boldsymbol{P}^{\text{in}}_\pm = \frac{\varepsilon_0\omega_0}{2k}|A^{\text{in}}_\pm|^2\hat{\boldsymbol{z}}, \tag{S8}$$

where the momentums of the two independent branches are respectively

$$\boldsymbol{P}^\sigma_\pm = \delta^\sigma \frac{|A^{\text{in}}_\pm|^2}{8k\varepsilon_1(t_0)\dot{\psi}_\pm(t_0)} \left[\Big(\varepsilon_0\omega_0 - \delta^\sigma\varepsilon_1(t_0)\dot{\psi}_\pm(t_0)\Big)^2 + \left(k\big(\Theta_1(t_0) - \Theta_0\big) - \varepsilon_1(t_0)\frac{\dot{\rho}_\pm(t_0)}{\rho_\pm(t_0)}\right)^2\right]\hat{\boldsymbol{z}}, \quad (\sigma = 1, 2). \tag{S9}$$

Fig. S1 shows the momentums for the simplified case discussed in the main text. We can see that the momentums of the two parts are always in opposite directions. Supposing $\dot{\psi}_\pm(t_0) < 0$, then $\boldsymbol{P}^1_\pm$ is always along the incident direction while $\boldsymbol{P}^2_\pm$ is along the inverse direction, and their vector sum always equals to the incident momentum. Therefore, $\boldsymbol{B}^1_\pm$ and $\boldsymbol{B}^2_\pm$ have clear physical meaning, i.e. the time refraction and the time reflection of the corresponding circularly polarized incident wave.

Similarly, we can calculate the Poynting vectors $\boldsymbol{S}_\pm = \mathfrak{Re}(\boldsymbol{E}^1_\pm + \boldsymbol{E}^2_\pm) \times \mathfrak{Re}(\boldsymbol{H}^1_\pm + \boldsymbol{H}^2_\pm)$ of the TCB modes. It can be demonstrated that the cross term of the time refracted and reflected parts also vanishes for each TCB mode: $\boldsymbol{S}^{\text{cross}}_\pm = \mathfrak{Re}(\boldsymbol{E}^1_\pm) \times \mathfrak{Re}(\boldsymbol{H}^2_\pm) + \mathfrak{Re}(\boldsymbol{E}^2_\pm) \times \mathfrak{Re}(\boldsymbol{H}^1_\pm) = 0$. Thus the total energy flow equals to the sum of the time

refracted and reflected parts:

$$\boldsymbol{S}_{\pm} = \boldsymbol{S}_{\pm}^{1} + \boldsymbol{S}_{\pm}^{2}, \tag{S10}$$

where

$$\boldsymbol{S}_{\pm}^{\sigma} = \mathfrak{Re}(\boldsymbol{E}_{\pm}^{\sigma}) \times \mathfrak{Re}(\boldsymbol{H}_{\pm}^{\sigma}) = \frac{1}{\varepsilon_1(t)\mu_1(t)}\boldsymbol{P}_{\pm}^{\sigma} \quad (\sigma = 1, 2). \tag{S11}$$

Therefore, the Poynting vectors change with time in general, and $\boldsymbol{S}_{\pm} \neq \boldsymbol{S}_{\pm}^{\mathrm{in}}$ unless $\varepsilon_1\mu_1 \equiv \varepsilon_0\mu_0$. In addition, the energy densities corresponding to the refracted and reflected parts are, respectively,

$$W_{\pm}^{\sigma} = \frac{1}{2}\Big[\mathfrak{Re}(\boldsymbol{D}_{\pm}^{\sigma}) \cdot \mathfrak{Re}(\boldsymbol{E}_{\pm}^{\sigma}) + \mathfrak{Re}(\boldsymbol{B}_{\pm}^{\sigma}) \cdot \mathfrak{Re}(\boldsymbol{H}_{\pm}^{\sigma})\Big] = \frac{|A_{\pm}^{\sigma}|^2}{4}\left[\frac{\varepsilon_1(t)}{k^2}\dot{\rho}_{\pm}(t)^2 + \left(\frac{\varepsilon_1(t)\dot{\psi}_{\pm}(t)^2}{k^2} + \frac{1}{\mu_1(t)}\right)\rho_{\pm}(t)^2\right]. \tag{S12}$$

However, the cross term $W_{\pm}^{\mathrm{cross}}$ of the refracted and reflected parts does not equal to zero, so $W_{\pm} \neq W_{\pm}^{1} + W_{\pm}^{2}$.

It should be noted that the definition of time refracted and reflected parts is not unique, because we can arbitrarily choose the pair of independent real solutions $g(t)$ and $h(t)$ given in Eq. (S3). How to define the time refraction and reflection uniquely is still an open question in general situations. Nevertheless, to choose the pair of plane wave solutions $T_{\pm}^{\sigma} = \mathrm{e}^{\mathrm{i}\delta^{\sigma}\omega_{\pm}t}$ as the time refraction and reflection seems quite reasonable in the simplified case discussed in the main text. In the situation of Gaussian pulse incidence, this choice ensures the defined refracted pulse and reflected pulse move in opposite directions with group velocities (see main text for details).

For the simplified case, the Poynting vectors and energy densities take the form

$$\boldsymbol{S}_{\pm}^{\sigma} = \langle \boldsymbol{S}_{\pm}^{\sigma} \rangle = -\delta^{\sigma}\frac{\omega_{\pm}}{2k\mu_1}|A_{\pm}^{\sigma}|^2\hat{\boldsymbol{z}}, \tag{S13a}$$

$$W_{\pm}^{\sigma} = \langle W_{\pm}^{\sigma} \rangle = \frac{k \pm \beta/2}{2k\mu_1}|A_{\pm}^{\sigma}|^2. \tag{S13b}$$

Therefore, the energy transport velocities of the two TCB modes are determined by

$$\boldsymbol{v}_{\mathrm{E}\pm} = \frac{\langle \boldsymbol{S}_{\pm} \rangle}{\langle W_{\pm} \rangle} = v_1\frac{\sqrt{k(k \pm \beta)}}{k \pm \beta/2}\hat{\boldsymbol{k}}. \tag{S14}$$

As we have discussed in the main text, the energy is not conserved generically in time-dependent systems. However, $\boldsymbol{S}_{\pm}^{\sigma}$ and $W_{\pm}^{\sigma}$ given in Eqs. (S13) are both invariant with time. The reason of this exceptional conservation is that the axion coupling in the lagrangian density $\mathcal{L}_{\Theta} = \Theta(t)\boldsymbol{E}_{\pm}^{\sigma} \cdot \boldsymbol{B}_{\pm}^{\sigma} = 0$ for the pair of TCB plane-wave modes.

For ordinary spatial refraction and reflection, the conservation of energy leads to the equality of the incident light intensity $I^0 = |\langle \boldsymbol{S}^{\mathrm{in}} \rangle|$ with the sum of the transmitted intensity $I^T = |\langle \boldsymbol{S}^1 \rangle|$ and the reflected intensity $I^R = |\langle \boldsymbol{S}^2 \rangle|$: $I^0 = I^T + I^R$ (for one dimension), while this equality is false for time refraction and time reflection. If we follow the traditional definitions of transmissivity $T = I^T/I^0$ and reflectivity $R = I^R/I^0$, then $T + R \neq 1$ owing to energy non-conservation. However, we can introduce modified transmissivity and modified reflectivity respectively

$$T = \frac{I^T}{I^{T+R}} = \frac{|\langle \boldsymbol{S}^1 \rangle|}{|\langle \boldsymbol{S}^1 \rangle| + |\langle \boldsymbol{S}^2 \rangle|}, \quad R = \frac{I^R}{I^{T+R}} = \frac{|\langle \boldsymbol{S}^2 \rangle|}{|\langle \boldsymbol{S}^1 \rangle| + |\langle \boldsymbol{S}^2 \rangle|}. \tag{S15}$$

Under these redefinitions, the normalization condition is satisfied: $T + R = 1$ (see Fig. 2(a) in the main text).

Front velocity of TCB modes

Ref. [S1, S2] point out that the superluminal group velocities $v_{\mathrm{g}\pm}$ of CFJ modes indicate the violation of causality. However, our calculation in Eq. (S14) shows that the energy transport velocity is lower than the speed of light in vacuum, although, the energy transport velocity defined in Eq. (S14) is more "interpretive" than measurable [S3]. By contrast, a more visualized definition is the front velocity which gives the speed of the wavefront of a step-function discontinuous wave and measures the speed of information propagation [S4, S5]. In the following section, we will prove that the front velocity $v_{\mathrm{f}} \equiv v_1$ in the linearly varying axion-type media.

Firstly, let's give the Fourier expansion with respect to wave vector k for an arbitrary TM wave $\boldsymbol{B}(z,t)$ traveling along z axis in linearly varying axion-type ME media:

$$\begin{aligned}\boldsymbol{B}(z,t) = & \int_{-\infty}^{\infty} \mathrm{d}k \left(A_-^1(k)\mathrm{e}^{\mathrm{i}\omega_-(k)t} + A_-^2(k)\mathrm{e}^{-\mathrm{i}\omega_-(k)t} \right) \mathrm{e}^{\mathrm{i}kz}\, \hat{\boldsymbol{U}}_+ \\ & + \int_{-\infty}^{\infty} \mathrm{d}k \left(A_+^1(k)\mathrm{e}^{\mathrm{i}\omega_+(k)t} + A_+^2(k)\mathrm{e}^{-\mathrm{i}\omega_+(k)t} \right) \mathrm{e}^{\mathrm{i}kz}\, \hat{\boldsymbol{U}}_- .\end{aligned} \tag{S16}$$

where the piecewise "frequencies" are

$$\omega_-(k) = \begin{cases} v_1 k\sqrt{(k-\beta)/k} & k \in (-\infty, 0) \cup (\beta, \infty) \\ \mathrm{i}v_1 k\sqrt{(-k+\beta)/k} & k \in [0, \beta] \end{cases} \tag{S17a}$$

$$\omega_+(k) = \begin{cases} v_1 k\sqrt{(k+\beta)/k} & k \in (-\infty, -\beta) \cup (0, \infty) \\ -\mathrm{i}v_1 k\sqrt{(-k-\beta)/k} & k \in [-\beta, 0] \end{cases} \tag{S17b}$$

and they obey the relation $\omega_+(-k)^* = -\omega_-(k)$. The corresponding piecewise "phase velocities" are $v_\pm(k) = \omega_\pm(k)/k$. Because $\boldsymbol{B}$ is a real vector field: $\boldsymbol{B}(z,t) = \boldsymbol{B}(z,t)^*$, the Fourier coefficients are not independent:

$$A_-^\sigma(-k)^* = A_+^\sigma(k), \qquad (\sigma = 1, 2). \tag{S18}$$

Therefore, Eq. (S16) can be written as

$$\boldsymbol{B}(z,t) = \int_{-\infty}^{\infty} \mathrm{d}k \left(A_-^1(k)\mathrm{e}^{\mathrm{i}\omega_-(k)t} + A_-^2(k)\mathrm{e}^{-\mathrm{i}\omega_-(k)t} \right) \mathrm{e}^{\mathrm{i}kz}\, \hat{\boldsymbol{U}}_+ + \text{c.c.} \tag{S19}$$

We also can adopt another convention to define the piecewise "frequencies" and the piecewise "phase velocities":

$$\omega'_\pm(k) = \omega_\pm(k)^*, \quad v'_\pm(k) = v_\pm(k)^*. \tag{S20}$$

Here, we use symbols with prime, *e.g.* $\omega'_\pm(k)$ and $v'_\pm(k)$, to represent the second convention to differentiate from the first convention given in Eq. (S17). The two conventions are obviously equivalent to each other, if the the following transform relations are satisfied:

$$\begin{cases} A'^\sigma_\pm(k) = A^\sigma_\pm(k) \quad (\sigma = 1, 2), & \pm\, k \in (-\infty, -\beta) \cup (0, \infty) \\ A'^1_\pm(k) = A^2_\pm(k), \;\; A'^1_\pm(k) = A^2_\pm(k), & \pm\, k \in [-\beta, 0]. \end{cases} \tag{S21}$$

Now we consider the time refraction and reflection of an incident pulse with two well-defined front edges $z = \pm a$ at the time interface $t_0 = 0$, i.e. $\boldsymbol{B}(z,0) = B(z,0)\hat{\boldsymbol{U}}_+ + \text{c.c.} = 0$ for $|z| > a$, and $B(z,0)$ is supposed to be a smooth function. Without loss of the generality, we still choose $\varepsilon_1 = \varepsilon_0$, $\mu_1 = \mu_0$, $\Theta_1(0) = \Theta_0$, and assume that the incident pulse is merely superposed by the plane waves traveling towards the positive direction of z axis:

$$\boldsymbol{B}^{\text{in}}(z,t) = \int_{-\infty}^{\infty} A^{\text{in}}(k)\mathrm{e}^{\mathrm{i}k(z-v_0 t)}\hat{\boldsymbol{U}}_+ + \text{c.c.} \quad (t < 0). \tag{S22}$$

where $A^{\text{in}}(k)$ is given by

$$A^{\text{in}}(k) = \int_{-\infty}^{\infty} \mathrm{d}z B(z,0)\mathrm{e}^{-\mathrm{i}kz} = \int_{-a}^{a} \mathrm{d}z B(z,0)\mathrm{e}^{-\mathrm{i}kz}. \tag{S23}$$

Since $B(z,0)$ is a smooth function with the compact support $[-a, a]$, according to the Paley-Wiener-Schwartz theorem [S6], $A^{\text{in}}(\kappa)$ is analytic on the complex plane and satisfies

$$|A^{\text{in}}(\kappa)| \le \frac{C\,\mathrm{e}^{a|\mathfrak{Im}(\kappa)|}}{1 + |\kappa|}, \tag{S24}$$

for some constant C.

According to the law of time refraction and time reflection for a particular wave vector given in Eq. (6) and Eq. (7) in the main text, we have

$$\begin{aligned}\boldsymbol{B}(z,t) &= \int_{-\infty}^{\infty} \mathrm{d}k\, \frac{1}{2} \left[\sum_{\sigma=1,2} \left(1 - \delta^{\sigma} \frac{v_0}{v_-(k)} \right) \mathrm{e}^{\mathrm{i}\delta^{\sigma} \omega_-(k) t} \right] A^{\mathrm{in}}(k)\, \mathrm{e}^{\mathrm{i}kz}\, \hat{\boldsymbol{U}}_+ + \text{c.c.} \\ &= \sum_{\sigma=1,2} B^{\sigma}(z,t)\, \hat{\boldsymbol{U}}_+ + \text{c.c.} \qquad (t>0),\end{aligned} \tag{S25}$$

where

$$B^{\sigma}(z,t) = \int_{-\infty}^{\infty} \mathrm{d}k \frac{1}{2} \left(1 - \delta^{\sigma} \frac{v_0}{v_-(k)} \right) A^{\mathrm{in}}(k)\, \mathrm{e}^{\mathrm{i}k(z+\delta^{\sigma} v_-(k) t)} \qquad (\sigma = 1,2). \tag{S26}$$

Note that the similar expression $B'^{\sigma}(z,t)$ is also valid for the second convention given in Eq. (S20), and

$$\sum_{\sigma=1,2} B^{\sigma}(z,t) = \sum_{\sigma=1,2} B'^{\sigma}(z,t). \tag{S27}$$

To extend the integral (S26) to complex plane, we introduce two pairs of two-valued complex functions

$$u_{\pm}(\kappa) = v_1 \sqrt{(\kappa \pm \beta)/\kappa}, \qquad \Omega_{\pm}(\kappa) = \kappa\, u_{\pm}(\kappa) = v_1 \kappa \sqrt{(\kappa \pm \beta)/\kappa}, \tag{S28}$$

with complex variable $\kappa = k + \mathrm{i}\epsilon$. The two functions have two branch points $\kappa = 0$, $\kappa = \mp\beta$ on real axis, and the line segment between the two points is the branch cut. Note that to choose the branch cut in this way follows the single-valued branch of square root function: $\sqrt{\kappa} := \sqrt{|\kappa|}\mathrm{e}^{\mathrm{i}\arg(\kappa)/2}$ with the convention $\arg(\kappa) \in (-\pi, \pi]$. The limit from the upper half plane to real axis

$$\begin{aligned}\lim_{\epsilon\to 0+} u_{\pm}(k+\mathrm{i}\epsilon) &= \lim_{\epsilon\to 0+} v_1 \sqrt{\left(1 \pm \beta \frac{k}{k^2+\epsilon^2}\right) \mp \mathrm{i} \frac{\beta\epsilon}{k^2+\epsilon^2}} = \lim_{\epsilon\to 0+} v_1 \sqrt{\frac{k\pm\beta}{k} \mp \mathrm{i}\epsilon} \\ &= \begin{cases} v_1 \sqrt{(k\pm\beta)/k} & k \notin \text{Branch cut} \\ \mp \mathrm{i} \sqrt{(-k\mp\beta)/k} & k \in \text{Branch cut} \end{cases}\end{aligned} \tag{S29}$$

is exactly the piecewise "phase velocity", and the limit of $\Omega_{\pm}(\kappa)$ is accordingly the piecewise "frequency" under the first convention, i.e.

$$\lim_{\epsilon\to 0+} u_{\pm}(k+\mathrm{i}\epsilon) = v_{\pm}(k), \qquad \lim_{\epsilon\to 0+} \Omega_{\pm}(k+\mathrm{i}\epsilon) = \omega_{\pm}(k). \tag{S30}$$

On the contrary, the limits from the lower half plane to real axis gives the quantities under the second convention:

$$\lim_{\epsilon\to 0-} u_{\pm}(k+\mathrm{i}\epsilon) = v'_{\pm}(k), \qquad \lim_{\epsilon\to 0-} \Omega_{\pm}(k+\mathrm{i}\epsilon) = \omega'_{\pm}(k). \tag{S31}$$

Therefore, $B^{\sigma}(z,t)$ defined in the first convention can be written as a complex integral

$$\begin{aligned}B^{\sigma}(z,t) &= \int_{-\infty+\mathrm{i}0+}^{\infty+\mathrm{i}0+} \mathrm{d}\kappa\, \frac{1}{2} \left(1 - \delta^{\sigma} \frac{v_0}{u_-(\kappa)} \right) A^{\mathrm{in}}(\kappa) \mathrm{e}^{\mathrm{i}\kappa(z+\delta^{\sigma} u_-(\kappa) t)} \\ &= \left(\int_{\mathrm{I}} + \int_{\mathrm{II}} \right) \mathrm{d}\kappa\, \tilde{B}^{\sigma}(k,t) \mathrm{e}^{\mathrm{i}\kappa(z+\delta^{\sigma} v_1 t)},\end{aligned} \tag{S32}$$

and so do $B'^{\sigma}(z,t)$ defined in the second convention:

$$\begin{aligned}B'^{\sigma}(z,t) &= \int_{-\infty+\mathrm{i}0-}^{\infty+\mathrm{i}0-} \mathrm{d}\kappa\, \frac{1}{2} \left(1 - \delta^{\sigma} \frac{v_0}{u_-(\kappa)} \right) A^{\mathrm{in}}(\kappa) \mathrm{e}^{\mathrm{i}\kappa(z+\delta^{\sigma} u_-(\kappa) t)} \\ &= \left(\int_{\mathrm{I}'} + \int_{\mathrm{II}'} \right) \mathrm{d}\kappa\, \tilde{B}^{\sigma}(\kappa,t) \mathrm{e}^{\mathrm{i}\kappa(z+\delta^{\sigma} v_1 t)},\end{aligned} \tag{S33}$$

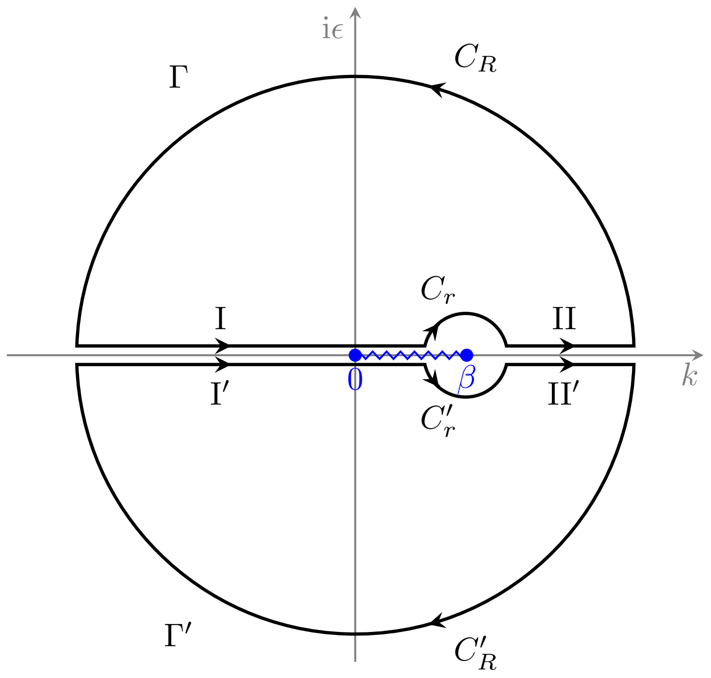

FIG. S2. Contour Γ and contour Γ' for the two integrals given in Eq. (S35). Contour Γ (Γ') can separate into four parts: I, II, C_r, and C_R (I′, II′, C'_r, and C'_R). $k=0$ and $k=\beta$ on the real axis are two branch points of $u_\pm(\kappa)$ and the integrand, their connecting line is the branch cut.

where $\tilde{B}^\sigma(\kappa,t)$ is short for

$$\tilde{B}^\sigma(\kappa,t)=\frac{1}{2}\left(1-\delta^\sigma\frac{v_0}{u_-(\kappa)}\right)A^{\mathrm{in}}(\kappa)\mathrm{e}^{\mathrm{i}\kappa\delta^\sigma(u_-(\kappa)-v_1)t}. \tag{S34}$$

The integrals need to be separated into I and II (or I′ and II′) two parts because the branch point $\kappa=\beta$ is also a pole of the integrand. The integrand is analytic in the whole complex plane except the branch cut on the real axis, so the contour integrals with either the contour Γ or the contour Γ' shown in Fig.S2 equal to zero

$$\oint_\Gamma \mathrm{d}\kappa\,\tilde{B}^\sigma(\kappa,t)\mathrm{e}^{\mathrm{i}\kappa(z+\delta^\sigma v_1 t)}=\left(\int_{\mathrm{I}}+\int_{\mathrm{II}}+\int_{C_r}+\int_{C_R}\right)\mathrm{d}\kappa\,\tilde{B}^\sigma(\kappa,t)\mathrm{e}^{\mathrm{i}\kappa(z+\delta^\sigma v_1 t)}=0, \tag{S35a}$$

$$\oint_{\Gamma'} \mathrm{d}\kappa\,\tilde{B}^\sigma(\kappa,t)\mathrm{e}^{\mathrm{i}\kappa(z+\delta^\sigma v_1 t)}=\left(\int_{\mathrm{I}'}+\int_{\mathrm{II}'}+\int_{C'_r}+\int_{C'_R}\right)\mathrm{d}\kappa\,\tilde{B}^\sigma(\kappa,t)\mathrm{e}^{\mathrm{i}\kappa(z+\delta^\sigma v_1 t)}=0, \tag{S35b}$$

where C_r is an infinitesimal semicircle with radius r above the pole $k=\beta$, C_R is an infinite semicircle with radius R in the upper half plane, and C'_r, C'_R are their counterparts in the lower half plane.

For the third term of Eq. (S35a),

$$\begin{aligned}
&\left|\int_{C_r}\mathrm{d}\kappa\,\frac{1}{2}\left(1-\delta^\sigma\frac{v_0}{u_-(\kappa)}\right)A^{\mathrm{in}}(\kappa)\mathrm{e}^{\mathrm{i}\kappa(z+\delta^\sigma u_-(\kappa)t)}\right|\\
=&\left|\int_\pi^0 r\mathrm{e}^{\mathrm{i}\phi}\mathrm{i}\mathrm{d}\phi\,\frac{1}{2}\left(1-\delta^\sigma\sqrt{\frac{r\mathrm{e}^{\mathrm{i}\phi}+\beta}{r\mathrm{e}^{\mathrm{i}\phi}}}\right)A^{\mathrm{in}}(r\mathrm{e}^{\mathrm{i}\phi}+\beta)\mathrm{e}^{\mathrm{i}(r\mathrm{e}^{\mathrm{i}\phi}+\beta)(z+\delta^\sigma u_-(r\mathrm{e}^{\mathrm{i}\phi}+\beta)t)}\right|\\
\le&\frac{\pi r}{2}\max\left[\left(1-\delta^\sigma\sqrt{\frac{r\mathrm{e}^{\mathrm{i}\phi}+\beta}{r\mathrm{e}^{\mathrm{i}\phi}}}\right)A^{\mathrm{in}}(r\mathrm{e}^{\mathrm{i}\phi}+\beta)\mathrm{e}^{\mathrm{i}(r\mathrm{e}^{\mathrm{i}\phi}+\beta)(z+\delta^\sigma u_-(r\mathrm{e}^{\mathrm{i}\phi}+\beta)t)}\right]\\
\le&\frac{\pi r}{2}\max\left|1-\delta^\sigma\sqrt{\frac{r\mathrm{e}^{\mathrm{i}\phi}+\beta}{r\mathrm{e}^{\mathrm{i}\phi}}}\right|\max\left|A^{\mathrm{in}}(r\mathrm{e}^{\mathrm{i}\phi}+\beta)\mathrm{e}^{\mathrm{i}(r\mathrm{e}^{\mathrm{i}\phi}+\beta)(z+\delta^\sigma u_-(r\mathrm{e}^{\mathrm{i}\phi}+\beta)t)}\right|,
\end{aligned} \tag{S36}$$

where

$$\max\left|1-\delta^{\sigma}\sqrt{\frac{re^{i\phi}+\beta}{re^{i\phi}}}\right|\leq\max\left(1+\sqrt{\left|\frac{re^{i\phi}+\beta}{re^{i\phi}}\right|}\right)=1+\sqrt{\frac{r+\beta}{r}}, \tag{S37}$$

and $A^{\rm in}(re^{i\phi}+\beta)e^{i(re^{i\phi}+\beta)(z+\delta^{\sigma}u_{-}(re^{i\phi}+\beta)t)}$ is bounded as $r\to 0$, therefore we have

$$\lim_{r\to 0}\left|\int_{C_r}d\kappa\,\frac{1}{2}\left(1-\delta^{\sigma}\frac{v_0}{u_{-}(\kappa)}\right)A^{\rm in}(\kappa)e^{i\kappa(z+\delta^{\sigma}u_{-}(\kappa)t)}\right|=0. \tag{S38}$$

So the third term has no contribution to the contour integral.

For an integration $\int_{C_R}f(\kappa)d\kappa$ along the infinite semicircle C_R in the upper half plane, if $|zf(z)|$ tends to zero uniformly when $|z|\to\infty$ both in the upper half plane and on the real axis, then the integral will vanish. We thus need to check the limit of $|\kappa\tilde{B}^{\sigma}(\kappa,t)e^{i\kappa(z+\delta^{\sigma}v_1t)}|$ as $|\kappa|\to\infty$ for calculating the fourth term of Eq. (S35a). In light of Eq. (S24), we have the following inequality

$$\begin{aligned}\left|\kappa\tilde{B}(\kappa,t)e^{i\kappa(z+\delta^{\sigma}v_1t)}\right| =&|\kappa|\left|\frac{1}{2}\left(1-\delta^{\sigma}\frac{v_0}{u_{-}(\kappa)}\right)A^{\rm in}(\kappa)e^{i\kappa\delta^{\sigma}(u_{-}(\kappa)-v_1)t}e^{i\kappa(z+\delta^{\sigma}v_1t)}\right|\\ \leq&\frac{|\kappa|}{2}\left|\left(1-\delta^{\sigma}\frac{v_0}{u_{-}(\kappa)}\right)e^{i\kappa\delta^{\sigma}(u_{-}(\kappa)-v_1)t}\right|\frac{C\,e^{a\epsilon}}{1+|\kappa|}e^{-\epsilon(z+\delta^{\sigma}v_1t)}\\ =&\frac{C}{2}\left|\left(1-\delta^{\sigma}\frac{v_0}{u_{-}(\kappa)}\right)e^{i\kappa\delta^{\sigma}(u_{-}(\kappa)-v_1)t}\right|\frac{|\kappa|\,e^{-\epsilon(z-a+\delta^{\sigma}v_1t)}}{1+|\kappa|}.\end{aligned} \tag{S39}$$

If $z-a+v_1t>z-a-v_1t>0$ for $t>0$, the exponential term tends to zero in the upper half plane. And on account of the limit $\lim_{|\kappa|\to\infty}u_{-}(\kappa)=v_1$, $|\kappa\tilde{B}^{\sigma}(\kappa,t)e^{i\kappa(z+\delta^{\sigma}v_1t)}|\to 0$ uniformly for $|\kappa|\to\infty$ both in the upper half plane and on the real axis. Thus the integral of the fourth term also vanishes when $R\to\infty$ as long as $z-a+v_1t>z-a-v_1t>0$. According to Eq. (S35a), the sum of the first two terms also should be zero as $z-a-v_1t>0$ $(t>0)$, then we obtain $B^{\sigma}(z,t)=0\quad(\sigma=1,2)$, for $z-a-v_1t>0$ and $t>0$. Substituting this result into Eq. (S25) yields

$$\boldsymbol{B}(z,t)=0,\quad \text{for } z>v_1t+a\ \ (t>0). \tag{S40}$$

A similar analysis of the integral given in Eq. (S35b) leads to the result:

$$\boldsymbol{B}(z,t)=0,\quad \text{for } z<-(v_1t+a)\ \ (t>0). \tag{S41}$$

In other words, the wavefronts of both front and back edges can not propagate with a speed faster than v_1.

On the other hand, if $|z|<v_1t+a$, no matter Γ or Γ' is chosen to calculate $B^{\sigma}(z,t)$ (or $B'^{\sigma}(z,t)$), at least one of $B^1(z,t)$ and $B^2(z,t)$ would not be zero. Therefore, $\boldsymbol{B}(z,t)\neq 0$ for $|z|<v_1t+a$ $(t>0)$. In conclusion, the velocity of wave front is exactly v_1.

In the above discussion, we only concern the dispersion caused by $\dot{\Theta}=\beta$. In practice, the permittivity ε_1 and permeability μ_1 have dispersion with k in media, and accordingly $v_1=1/\sqrt{\varepsilon_1\mu_1}$ is also some function of k. The front velocity thus depends on the analyticity of $v_1(k)$ for actual materials and need to be further investigated. However, for the interaction between light and time-dependent true axion field in vacuum, or for the CFJ modes in Chern-Simons modified electrodynamics, the dispersion relation is precisely $\omega_{\pm}=ck\sqrt{(k\pm\beta)/k}$, so the front velocity $v_{\rm f}\equiv c$ and the causality will not be violated.

[S1] S. M. Carroll, G. B. Field, and R. Jackiw, Phys. Rev. D **41**, 1231 (1990).
[S2] Y. Itin, Phys. Rev. D **70**, 025012 (2004); Gen. Relativ. Gravit. **40**, 1219 (2008).
[S3] G. Diener, Phys. Lett. A **235**, 118 (1997).
[S4] L. Brillouin, *Wave propagation and group velocity* (Academic Press, 1960).
[S5] P. W. Milonni, *Fast light, slow light and left-handed light* (CRC Press, 2004).
[S6] E. Zeidler, *Quantum Field Theory I: Basics in mathematics and physics* (Springer, 2009).
